# Directional microwave emission from femtosecond-laser illuminated linear arrays of superconducting rings

**DOI:** 10.1038/s41598-023-44751-x

**Published:** 2023-10-23

**Authors:** Thomas J. Bullard, Kyle Frische, Charlie Ebbing, Stephen J. Hageman, John Morrison, John Bulmer, Enam A. Chowdhury, Michael L. Dexter, Timothy J. Haugan, Anil K. Patnaik

**Affiliations:** 1 Air Force Research Laboratory, Wright Patterson AFB, OH 45433-7251 USA; 2 UES, Inc., Dayton, OH 45432 USA; 3 Air Force Institute of Technology, Wright Patterson AFB, OH 45433-7765 USA; 4 The Ohio State University, Columbus, OH 43210 USA; 5 UDRI-University of Dayton Research Institute, Dayton, OH 45469 USA; 6 National Research Council, Washington, DC 20001 USA

**Keywords:** Electrical and electronic engineering, Superconducting devices, Photonic devices

## Abstract

We examine the electromagnetic emission from two photo-illuminated linear arrays composed of inductively charged superconducting ring elements. The arrays are illuminated by an ultrafast infrared laser that triggers microwave broadband emission detected in the 1–26 GHz range. Based on constructive interference from the arrays a narrowing of the forward radiation lobe is observed with increasing element count and frequency demonstrating directed GHz emission. Results suggest that higher frequencies and a larger number of elements are achievable leading to a unique pulsed array emitter concept that can span frequencies from the microwave to the terahertz (THz) regime.

## Introduction

The microwave (1–300 GHz) and terahertz (~ 0.3–30 THz) spectral regimes are some of the most technologically transformative regions of the electromagnetic spectrum. However, despite a tremendous amount of study in the past 20–30 years, the THz regime is only partially explored^1^. This deficiency is driven by the fact that THz frequencies lie in the transition region between classical electromagnetics and quantum photonics limiting the power and efficiency of emitters. Of the few sources available photoconductive antennas (PCAs) are arguably the most well-established due to ease of use, reliability, and low cost^2^. However, PCAs tend to be limited to frequencies below 3 THz, and while the strength of the generated field can be controlled by the voltage bias across the gap, the field is ultimately limited by dielectric breakdown and saturation effects^1,2^. One approach to overcome these limitations is to assemble an array^3–6^, which increases the gain of the ensemble of emitters. However, the array structure can introduce an additional set of limitations. For instance, photoconductive antennas must be directly wired to a voltage source that limits the application space. This requirement introduces weight and ohmic heating as well as unwanted electromagnetic coupling limiting the efficiency and performance of the array. Therefore, we explore an alternative approach for a high-fidelity emitter.

It is well established that when a current carrying superconductor is pulsed with an ultrafast laser with photon energy greater than the superconducting energy gap, THz electromagnetic radiation is generated with the associated electric field (E-field) proportional to the time rate of change of the current. Experimentally, this effect has been demonstrated with constant current biased YBa_2_Cu_3_O_7−x_ (YBCO) microbridges pulsed with a femtosecond 800 nm laser^7,8^. The radiation is attributed to the Cooper pair breaking and recombination process that occurs on the order of a picosecond^9–11^. This photo-response may in turn initiate several other non-equilibrium processes such as fast voltage transients^9–11^, photonic flux nucleation^12–16^, vortex motion^17,18^, superconducting gap suppression due to nonequilibrium electron heating^19^, and finally bolometric heating^20^. These processes may contribute to microwave radiation, which has also been detected when a current carrying superconductor is pulsed with an ultrafast infrared laser. For instance, a YBCO bridge was integrated into a constant current biased circuit connected to a normal metal antenna and pulsed with an ultrafast laser^21^. Microwave radiation due to bolometric processes was detected in addition to higher frequency emission associated with the faster Cooper pair breaking dynamics^21,22^.

In the prior examples, electromagnetic emission is obtained by directly wiring the superconductor to a feed circuit; however, an alternative approach exists for powering an array. Microwave radiation has been detected when a superconducting annulus carrying a persistent current is pulsed by an ultrafast laser^23–25^. The behavior of the annulus is similar to that of a resonant loop antenna with the laser illumination point breaking the azimuthal symmetry of the ring, playing a similar role to the voltage feed point in a normal metal antenna. However, coherent radiation from an ensemble of these annuli has not yet been investigated. Similarly, THz radiation has been detected from a YBCO thin film bow-tie antenna with super-currents circulating around a hole located at its geometric center^26^. As in the case of wired microbridges, the induced E-field emitted from the annuli and the bowtie is polarized parallel to the direction of the current at the location of laser illumination, and the sign of the E-field is reversed by reversing the direction of the current. These two facts suggest that if several regularly spaced superconducting annuli, each carrying a persistent current circulating in the same direction, are illuminated at the same relative location *the emitted E-field from each element will add coherently*.

To examine this hypothesis, we measure the radiative output from several superconducting annuli. These annuli are composed of patterned YBCO thin films deposited on sapphire substrates. Sample geometries are described in Table 1. Samples 1 and 2 are single rings while samples 3 and 4 are linear arrays with element count n.

**Table 1:** Sample geometries are described in the table.

Sample #	OD (mm)	ID (mm)	*d* (mm)	*n*
1	5	3	–	1
2	10	8	–	1
3	5	3	6.25	4
4	2.2	1.4	3.25	8

Columns from left to right: sample number (#), outer diameter (OD), inner diameter (ID), center to center spacing of elements (*d*), number of elements in the array (*n*).

**Figure 1:**
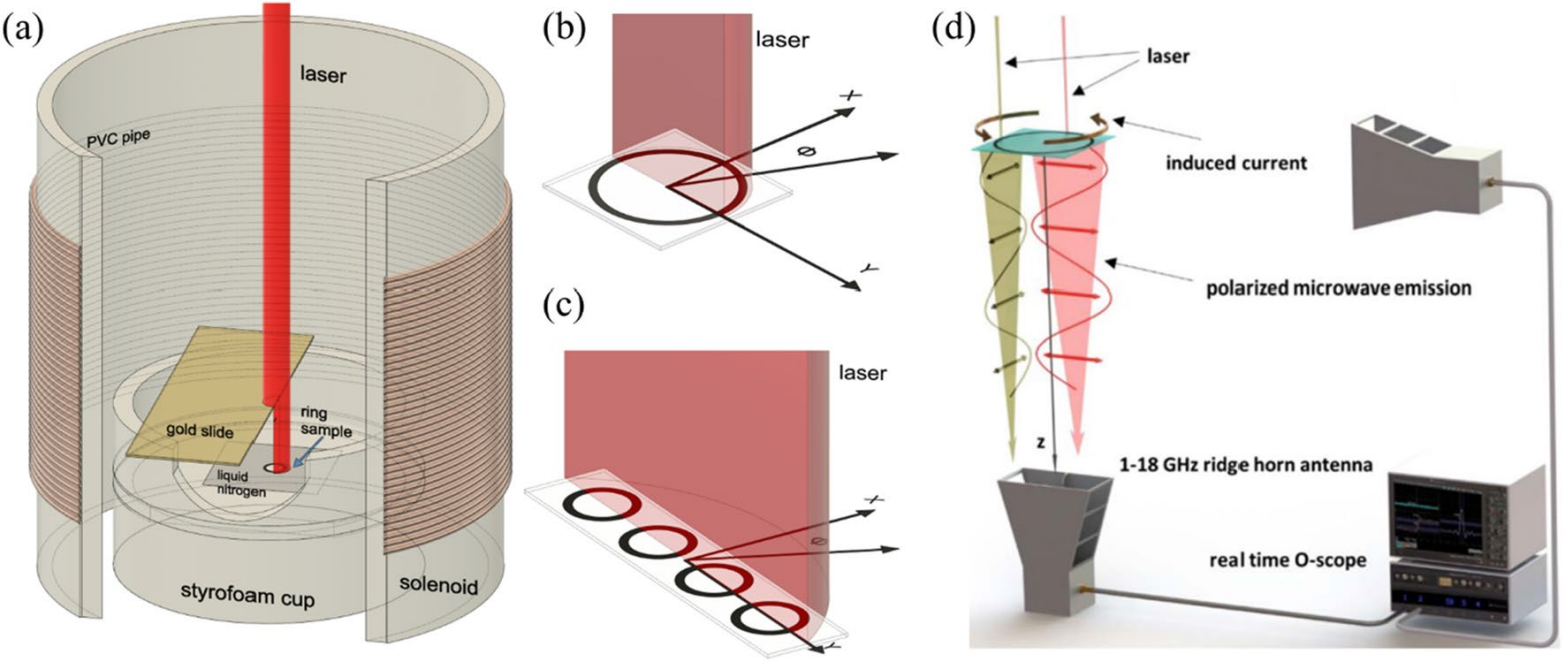
(**a**) Cutaway diagram of the experiment configuration. The sample sits inside a Styrofoam cup holding liquid nitrogen. The gold slide creates a shadow allowing a portion of the sample to be illuminated by the laser. (**b**) Half-illumination geometry for a single ring and (**c**) a four-element linear array. The x-axis points toward the in-plane antenna. (**d**) Notional diagram of the GHz-radiation measurement set-up^25^. Two antennas detect radiation from the sample. One is positioned in the plane of the ring, the other below the ring. Polarized radiation due to different laser illumination locations is indicated below the ring. (Note: diagrams are not drawn to scale).

We first measure the radiative output from a partially illuminated superconducting annulus carrying a persistent current induced by the magnetic field generated by a solenoid. A diagram of the measurement geometry is shown in Fig. 1a–d. By varying the position of a gold slide acting as a mask over the ring we obtain the illumination geometry that results in optimal radiative output. We then employ this geometry to examine the electromagnetic emission from two linear arrays composed of multiple superconducting rings. Specifically, we examine the radiation patterns for these arrays and compare it to the calculated pattern for the same geometry. As reported in the Results section, measured data and analytical predictions match well suggesting these arrays are a unique source of directional pulsed coherent microwave radiation. Further we consider these results a first step towards a unique directed coherent pulsed THz source.

## Results

In this section we present the results of systematically varying illumination conditions for a single annulus to determine the optimal forward radiating condition. The corresponding radiations patterns for this illumination condition are also obtained. We then use these results to study the directed maximal forward in-plane emission for two linear arrays of rings.

### Optimizing emission from a superconducting annulus

**Figure 2:**
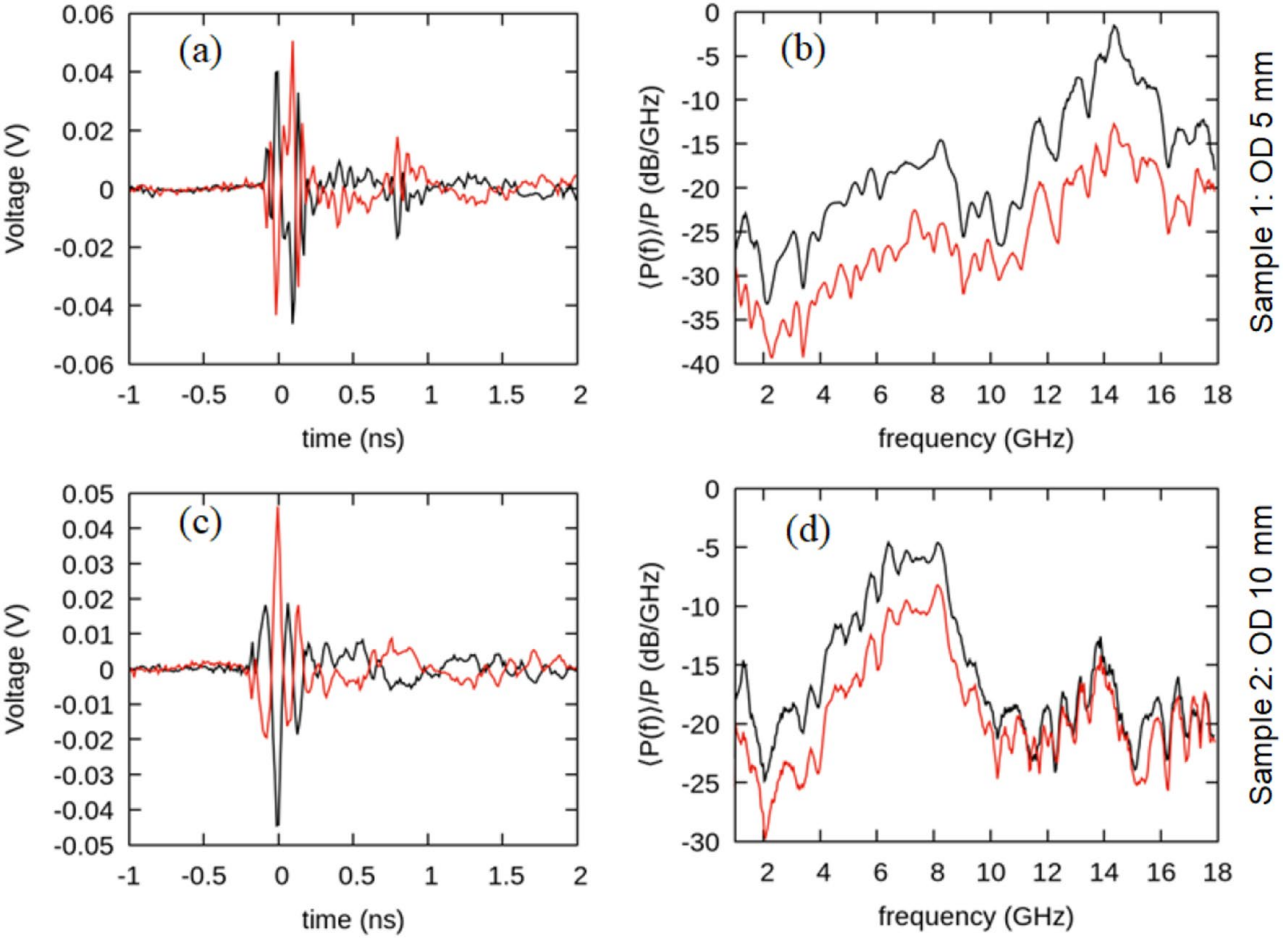
Uncalibrated single-pulse time domain signals measured in the plane of the ring are displayed in (**a**), and (**c**). The sign of the signals is flipped (indicated by the overlapping red and black curves) by illuminating opposite sides of the ring or reversing the circulation of the current in the ring. Associated normalized average power spectra (black) and standard deviation (red) are displayed in (**b**) and (**d**) for sample 1 (above) and sample 2 (below).

We first examine the radiative output from two single-ring elements. Typical uncalibrated time domain signals from samples 1 and 2 are shown in Fig. 2. The voltage–time signal reverses sign with the change in direction of the current. The associated normalized power spectra show frequency content that decreases with increasing ring diameter. We observe a dominant broad peak at ~ 14 GHz for sample 1 and ~ 7 GHz for sample 2 corresponding to resonant modes associated with the circumference of the ring as has been demonstrated in Ref.^25^.

**Figure 3:**
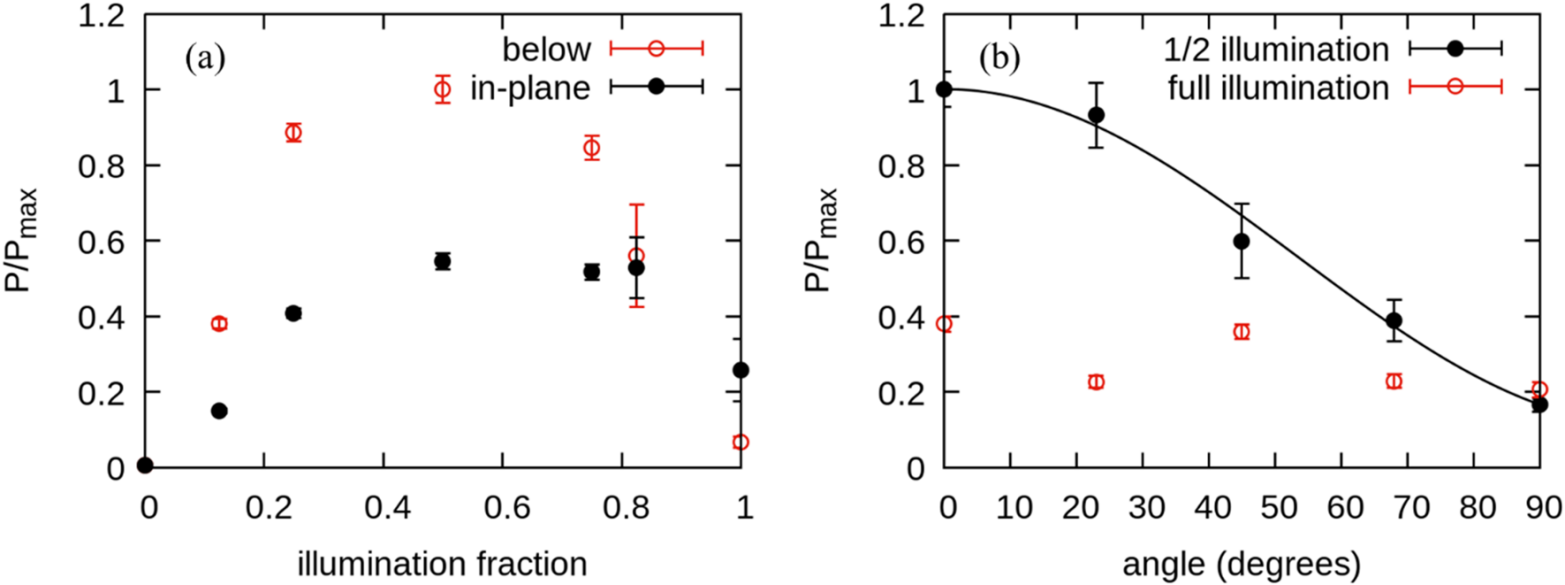
(**a**) Ring sample 2 is covered with a gold coated microscope slide mask and then progressively uncovered allowing more of the ring to be illuminated. The laser illumination spot has been centered such that it equally illuminates the entire ring when not blocked by the mask. Results are obtained below and in the plane of the ring. (**b**) Polarization curve of sample 1, half and fully illuminated. Power is measured below the ring. The fully illuminated ring does not appear to display any linear polarization. In both plots results are normalized to the maximum power in the plot ${P}_{max}$_._

The laser illumination geometry for the samples examined in Fig. 2 is shown in Fig. 1b. Specifically, half of the area of each ring is illuminated by the laser. This illumination condition is chosen to maximize radiation output. To demonstrate this, we examine different fractional illumination conditions for sample 1 and 2. We center the laser in the middle of the annulus and expand it such that the annulus is uniformly azimuthally illuminated. We then fully block the 800-nm laser light with the gold coated microscope slide. The slide is then incrementally translated allowing more laser light to illuminate the ring. Similar results are obtained using cardboard business cards acting as the laser mask. Power emitted from sample 2 is measured at each increment until the ring is fully illuminated, as shown in Fig. 3a. We do not observe a change in the distribution of the power in the spectrum as the illumination fraction changes. However, we do observe a change in the magnitude of the emitted power with the maximum occurring at half illumination. Further, in Fig. 3b we half-illuminate sample 1 and measure the polarization. The radiation detected below the ring is polarized parallel to the current at the center of the half-illuminated region. If the entire ring is illuminated, no selective polarization is detected as shown by the red data points in Fig. 3b.

As previously stated, illuminating half of the ring maximizes the detected power arriving at the Rx antennas. If we illuminate the half of the ring closest to the in-plane antenna, the induced E-field from the ring is polarized parallel to the Rx antennas. We infer that if we simultaneously illuminate the other half of the ring, it will contribute an antiparallel E-field component due to current moving in the opposite direction as demonstrated in Fig. 2a and c. Above and below the plane of the ring, these contributions result in destructive interference. In the plane of the ring the E-field from one side lags the E-field from the other side arriving at the antenna. This results in partially destructive interference at the in-plane Rx antenna. In support of this hypothesis, we note that in Fig. 3a the power detected below the ring at full illumination (illumination fraction = 1) is closer to zero than the power detected in the plane of the ring.

**Figure 4:**
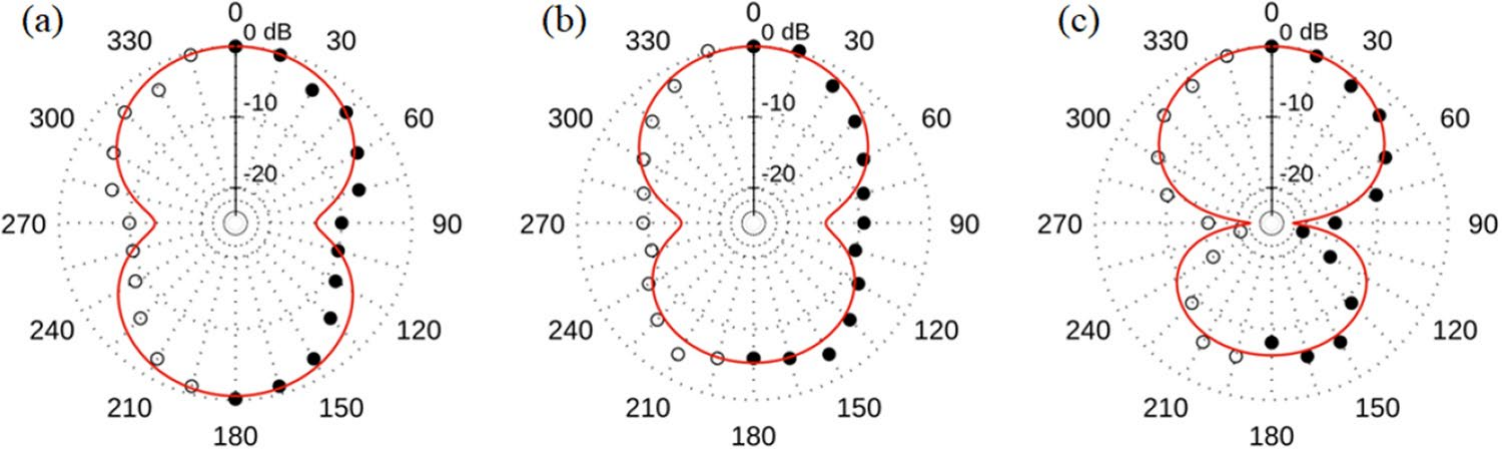
Radiation patterns for sample 1 measured at (**a**) 6.5 GHz, (**b**) 8.5 GHz, and (**c**) 14 GHz. The ring is half illuminated as shown in Fig. 1b. Black dots indicate normalized power measured at each angle. The red curve is a notional fit capturing the general shape of the radiation pattern. The sample sits at the center of the radiation pattern and is illuminated on the *ϕ* = 0° side of the ring. Data is measured from 0° to 180°. The mirror image is plotted as an aid to the eye. For the reference coordinate system see Fig. 1b.

Furthermore, we measure the radiation pattern for the half-illuminated sample 1. Results integrated in 500 MHz bands around frequencies 6.5, 8.5 and 14 GHz are shown in Fig. 4. We introduce fits to the data in the form of simple polar functions, indicated by the red curves, to capture the general shape of the radiation pattern. These notional fits are used in the calculation of the array radiation patterns in the next section. To the level of our approximation, we find that the normalized shape of the forward lobe (along *ϕ* = 0°) does not change with frequency, while the back lobe grows smaller. The smaller back lobe may be due to the gold slide being offset with respect to the ring. It has been demonstrated that the back lobe of a linear set of radiators can be suppressed by positioning the array asymmetrically with respect to a ground plane^27^.

### Superconducting annuli array: directional emission

Having measured the radiation pattern for a single ring element we now examine the emission from linear arrays composed of these elements. The in-plane field pattern of a linear array of isotropic elements is

1 $$h\left(\phi \right)=\frac{\mathrm{sin}\left(n\frac{\pi d}{\lambda }\mathrm{cos}\phi \right)}{n\cdot \mathrm{sin}\left(\frac{\pi d}{\lambda }\mathrm{cos}\phi\right)}$$

where $\lambda$ is the wavelength of interest and $\phi$ is the azimuthal angle measured in the plane of the array (see Fig. 1c). Thus, the in-plane radiation pattern is the square of the product of the array pattern and the individual element pattern $g\left(\phi \right)$ obtained from the measurement in the prior section^28^.

2 $$P\left(\phi \right)={\left|h\left(\phi \right)g\left(\phi \right)\right|}^{2}$$

**Figure 5:**
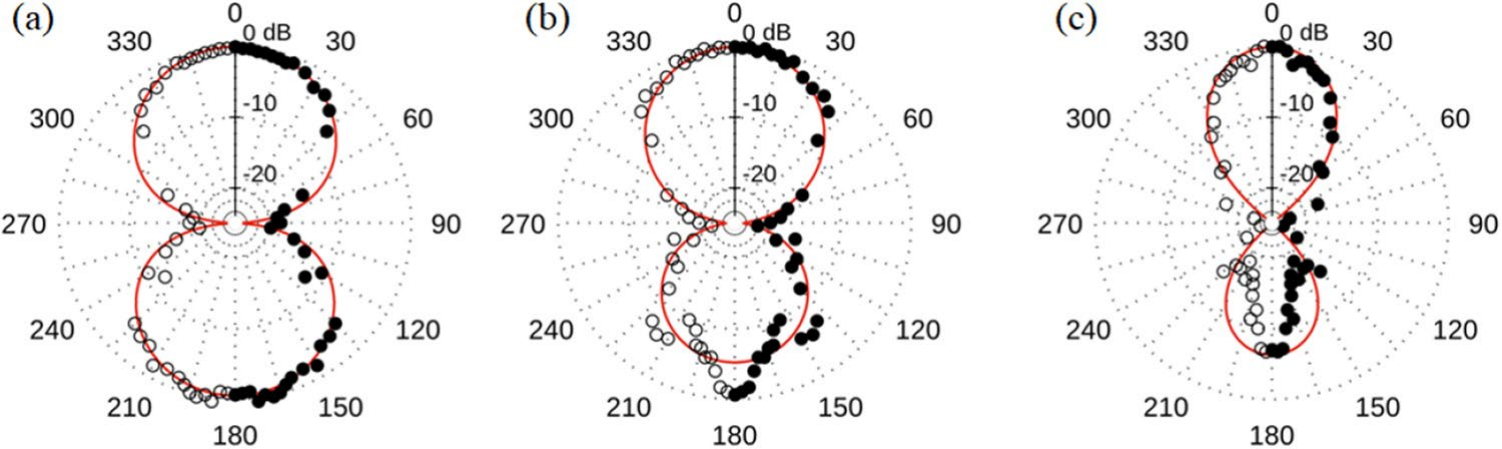
Radiation patterns for sample 3 (four-element array) at (**a**) 6.5 GHz, (**b**) 8.5 GHz, and (**c**) 14 GHz. A calculation for the radiation pattern based on Eqs. (1) and (2) is shown by the red curve. Black dots indicate normalized power measured at each angle. Data is measured from 0° to 180°. The mirror image is plotted as an aid to the eye. For the reference coordinate system see Fig. 1c.

**Figure 6:**
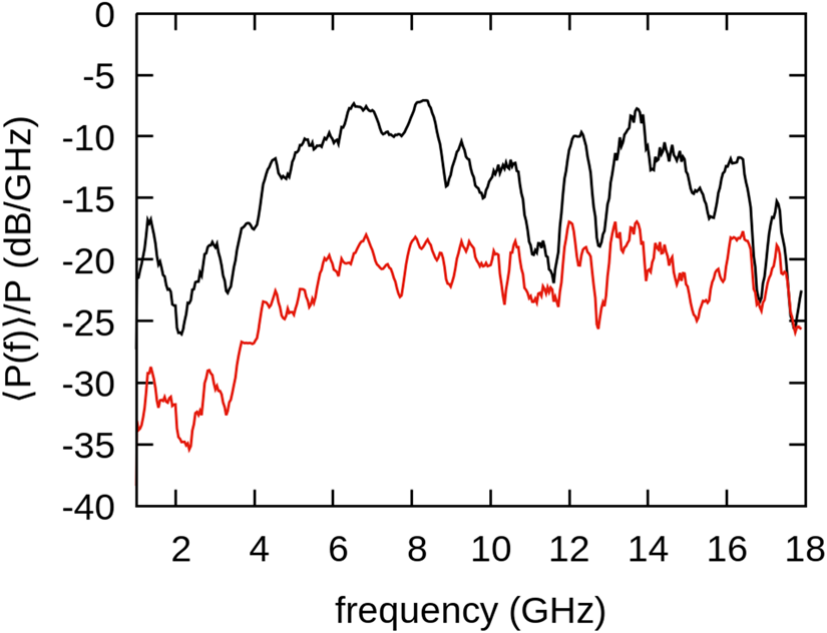
Normalized average power spectrum (black) and standard deviation (red) for the four-element array (sample 3) measured at *ϕ* = 0°.

To demonstrate coherent emission from array samples 3 and 4, we compare the measured radiation patterns to the calculated patterns based on Eqs. (1) and (2). Results for sample 3 (4 element array with 5 mm OD rings) are shown in Fig. 5. The spectrum captured along the $\phi ={0}^{^\circ }$ direction is shown in Fig. 6. We find major peaks at ~ 6.5 GHz, ~ 8.5 GHz and ~ 14 GHz in the spectrum. The ~ 14 GHz peak corresponds to the resonant frequency of the elements that comprise the array. However, we are currently unsure of the cause of the broad lower frequency content (2–11 GHz) in the spectrum. We hypothesize that the lower frequencies may correspond to array elements coupling to each other or to the larger dimensions of the array structure. In further studies we plan to vary the element spacing and the substrate size. This will yield additional insight as to whether this is indeed the case. An additional 12 GHz peak is present due to resonance with the charging coil as shown in the Supplementary Information. To obtain the radiation patterns we integrate in 500 MHz bands around the major peaks. As can be seen in Fig. 5 the in-plane − 3 dB beamwidth ($BW$) of the forward lobe decreases with frequency as predicted by Eqs. (1) and (2). At 6.5 GHz, $BW\sim {68}^{^\circ }$ while at 8.5 GHz, $BW\sim {58}^{^\circ }$, and at 14 GHz the forward lobe has narrowed to $BW\sim {41}^{^\circ }$.

**Figure 7:**
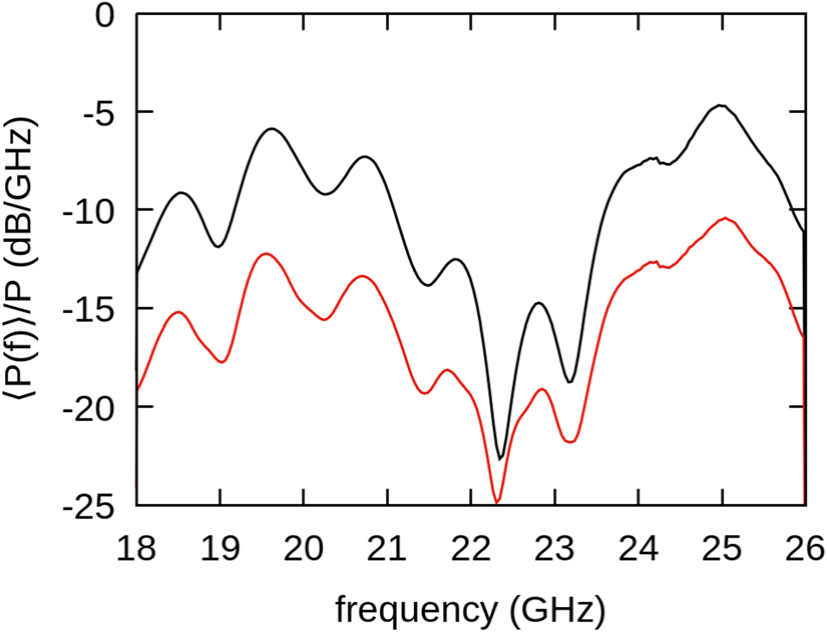
Normalized average power spectrum (black) and standard deviation (red) for the eight-element array (sample 4) measured at $\phi ={0}^{^\circ }$.

**Figure 8:**
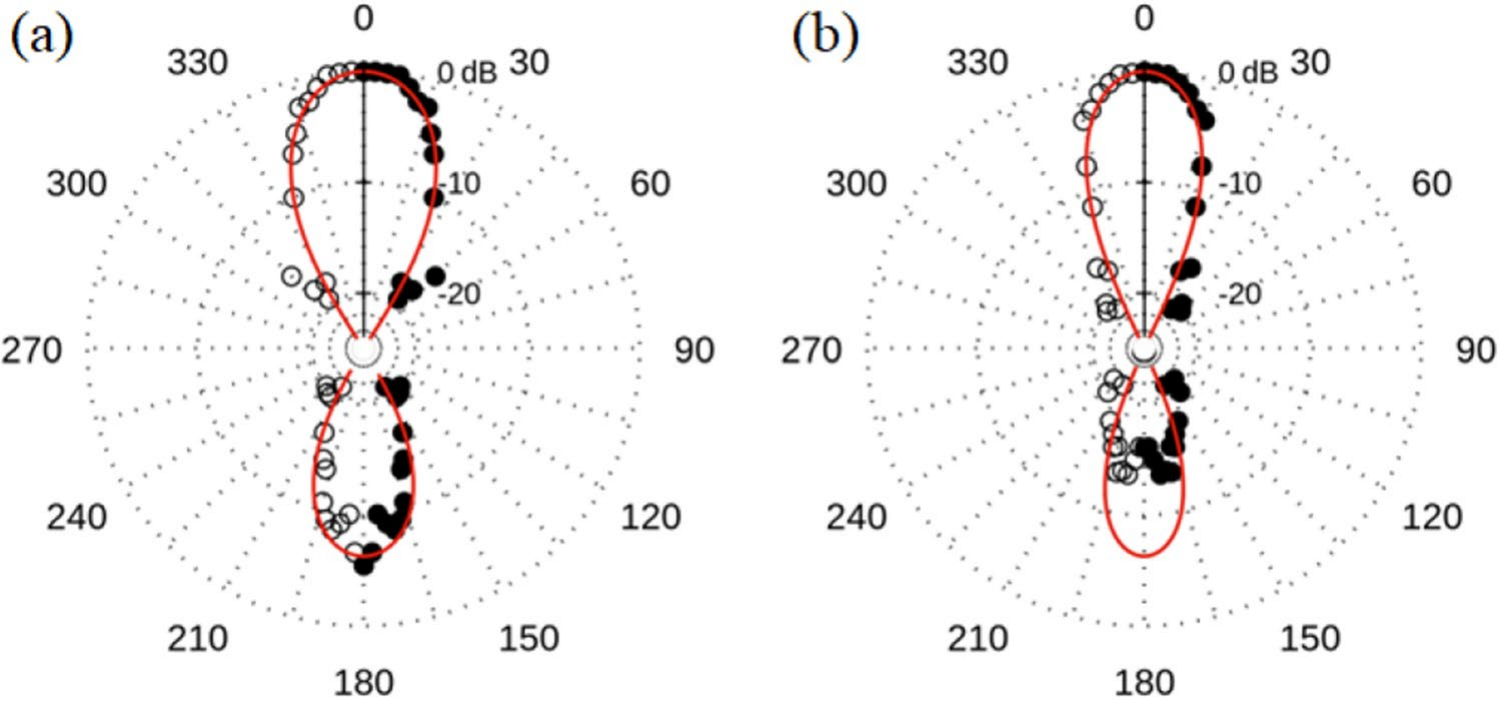
Radiation patterns for the eight-element array (sample 4) at (**a**) 19.5 GHz and (**b**) 25 GHz. The calculated radiation pattern based on Eqs. (1) and (2) is shown by the red curve. Black dots indicate normalized power measured at each angle. Data is measured from 0° to 180°. The mirror image is plotted as an aid to the eye. For the coordinate reference system see Fig. 1c.

Results for the 8 element array with 2.2 mm OD ring elements (sample 4) are shown in Figs. 7 and 8. The spectrum shows dominant peaks at ~ 19.5 and 25 GHz. Therefore, we integrate in two bands around these peaks (19–20 GHz and 24.5–25.5 GHz) to obtain the radiation patterns in Fig. 8a and b. We are unable to measure the radiation for a single 2.2 mm OD annulus as the signal is below our detection threshold. Therefore, to calculate $P\left(\phi \right)$ we use the radiation pattern of sample 2 at 14 GHz as a surrogate for our single element pattern $g\left(\phi \right)$. As previously noted, the normalized shape of the forward lobe for the individual ring does not change with frequency; however, the back lobe does reduce in size. This accounts for the difference between the data and the larger prediction of the back lobe in Fig. 8b. Measured forward $BW$ for 19.5 GHz is $\sim {30}^{^\circ }$ and $\sim {24}^{^\circ }$ for 25 GHz. For both sets of arrays we note that *the forward lobe matches the predicted general shape and narrows with higher frequency and number of elements, while the back lobe decreases in size commensurate with the single element measurements*.

**Figure 9:**
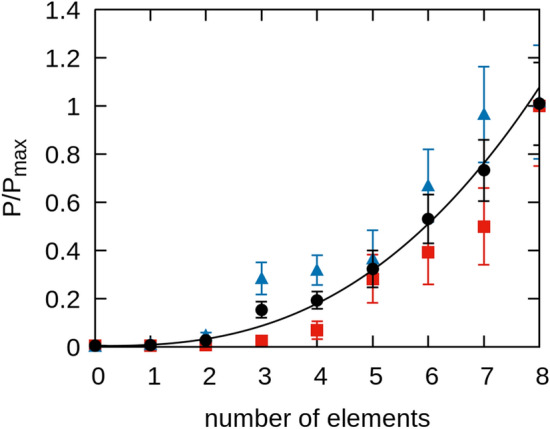
Power emitted from the eight-element array (sample 4). The power follows approximately quadratic behavior as the number of elements increases indicated by the curve. Blue and red data indicate separate series of measurements uncovering the rings from left to right vs. right to left respectively. The black datapoints indicate the mean of the left and right measurements. The estimated uncertainty is propagated from the standard deviation values associated with the left and right averages. Results are normalized to ${P}_{max}$, the mean power calculated for 8 elements.

We obtain further evidence of constructive interference by fully screening off all the elements of sample 4 from the laser and then progressively removing the screen. We measure the resulting emitted power as each larger set of elements is half-illuminated. The integrated power is captured below the array and in the 18–26 GHz band. We repeated this measurement twice, progressively uncovering the rings from left to right and then right to left to address any bias due to the positioning of the screen by eye. The two sets of measurements and the average are reported Fig. 9. We find that the average detected power increases with the number of elements as $P\propto {n}^{m}$ with $m=2.6\pm 0.1$. Along directions of constructive interference, the E-field of $n$ identical in-phase emitters add linearly by superposition such that the E-field is proportional to $n$. Therefore, the associated emitted power from the array will increase as $P\propto {n}^{2}$. The exponent obtained by our measurement suggests that constructive interference is occurring, and hence coherent radiation is being emitted. A similar analysis is performed in characterizing coherent THz radiation emitted from Bi_2_Sr_2_CaCu_2_O_8_ (BSCCO) mesas^29^.

## Summary and discussion

We have investigated the radiation emitted from two optically activated microwave arrays each composed of a line of equally spaced superconducting rings. The geometry of our emitter is unique compared to more traditional emitters such as PCAs. In our case, an external magnetic field is used to induce a persistent current in the superconducting ring elements eliminating the need for a directly connected feed circuit, which minimizes the weight, heat, and complexity on the array substrate. We find that half illumination of the rings by the laser maximizes radiative output. To realize this illumination condition, we use a mask positioned above the sample consisting of a gold-plated glass slide. Alternative masking options will be investigated elsewhere as well as optical solutions that could achieve a similar illumination condition. We attribute the diminished radiative emission when a ring is fully illuminated to destructive interference from emission from opposite sides of the ring. In future studies we plan to illuminate the ring at two points simultaneously with the laser and vary the relative location of the points on the ring. If our hypothesis is correct, we should observe destructive interference when opposite points on the ring are illuminated.

Furthermore, we have demonstrated that the measured in-plane radiation patterns associated with the arrays match the calculated forward lobe for a broadside collinear array for different frequencies and number of elements. We note that the radiation patterns were measured from $\phi ={0}^{^\circ }-{180}^{^\circ }$ due to limitations of the experimental configuration. However, we believe that the pattern is mirror symmetric about the y-axis due to the apparent goodness of fit to the predicted radiation pattern. We observe that *the inferred symmetric forward lobe narrows with increasing frequency and element count while the back lobe decreases with increasing frequency*. The difference of the front and back lobe suggests that the full 3d radiation pattern at higher frequencies differs from the radiation pattern of a collinear array composed of ideal dipole elements. The full radiation pattern might be similar to that of a fan beam with narrow beamwidth in the plane of the array and wide but directional beamwidth out of the plane of the array. Additional measurements will confirm whether this is in fact the case.

We also find that as the number of laser-illuminated elements increases the emitted power increases as a power law with exponent *m* = 2.6. This result suggests that, at a minimum, constructive interference is occurring where *m* = 2. This result further indicates that the elements with current circulating in the same direction are in-phase when simultaneously illuminated by the laser at the same relative location. Taken together these results demonstrate a unique pulsed coherent microwave emitter. With smaller radiating elements these arrays could provide an alternative coherent pulsed THz source.

While the exact cause of the emitted microwave radiation reported in this manuscript needs further investigation, we suspect that fast vortex motion plays a significant role. Magnetic flux lines must escape or enter the superconducting ring when pulsed by the laser and hence traverse the track width of the annulus (the difference between the outer and inner radius). In support of this idea, we note that microwave radiation generated by vortex flow crossing a superconducting/insulator interface has been demonstrated^30^ and is hypothesized to play a role in THz emission from BSCCO mesas^31^. In these cases, the emitted radiation occurs in a narrow frequency band and, therefore, the spectrum is not quite analogous to the broader emission observed from the annuli. Lengthening the pulse time of the laser beyond 1 ps will help to determine whether the faster Cooper pair breaking/recombination process or slower processes are the major contributor. Additional work will investigate optically induced flux motion in superconducting annuli via magneto-optical imaging and its relationship to the emitted microwave and THz radiation.

## Methods

YBCO thin film annuli of thickness 200 nm were fabricated by Star Cryoelectronics via photolithography on 0.5 mm thick sapphire substrates. Sample geometries are described in Table 1. Each sample is mounted on a Styrofoam block in a Styrofoam container, cooled with liquid nitrogen up to the level of the sample, and illuminated by a Ti:sapphire laser (800 nm wavelength, 40 fs pulse time), as depicted in Fig. 1a. The cryogenic container material is chosen such that it is transparent to microwave radiation. The target is illuminated by laser pulses each with an energy on the order of 1 mJ. The shape of the spot illuminating the rings and arrays is shown in Fig. 1b and c. The laser spot in Fig. 1b is positioned at the center of each sample and expanded such that each ring is uniformly azimuthally illuminated. However, for most of the results reported here, the samples are half-illuminated by blocking half of the laser with a gold coated (100 nm) microscope slide positioned ~ 1 cm above the sample as shown in Fig. 1a. The choice of this specific illumination pattern is discussed in the Results section. To illuminate the linear arrays, the laser passes through a cylindrical lens creating an elliptical mode that is centered on and spans the long axis of the array, as shown in Fig. 1c.

The supercurrent is induced in the rings via a magnetic field generated perpendicular to the plane of the ring by a solenoid that sits around the cryogenic holder, as shown in Fig. 1a. The solenoid is connected to a dc power supply and a relay that switches the charging circuit in-sync with the laser pulsing at 1 Hz. The current in the solenoid generates a magnetic field of ~ 30 Oe at the position of the sample and induces a circulating screening current that maintains zero flux through the center of the ring. When pulsed with the laser, the screening current is temporarily interrupted allowing magnetic field into the center of the ring. When the solenoid is turned off a portion of the magnetic field is now trapped at the center of the ring with a persistent current circulating around the annulus. The circulating current is pulsed again, and the magnetic field can no longer be maintained at the center of the ring. Microwave pulses are detected each time the ring is pulsed by the laser. Signals from the superconducting annuli are obtained at the ~ 1 Hz laser repetition rate. The charging coil remains in place for the entire measurement. The effects of the coil on the electromagnetic emission are investigated in the Supplementary Information.

Microwave radiation from the samples is captured by two sets of calibrated receive (Rx) horn antennas: one positioned below and the other in the plane of the sample as shown in Fig. 1d. We employ Rx antennas that span two frequency ranges: 1–18 GHz and 18–40 GHz depending on the frequency content of the signal emitted from the sample. The antenna positioned in the plane of the ring has its boresight aligned along the x-axis (*ϕ* = 0°) indicated in Fig. 1b and c, unless specified otherwise. All measurements are taken such that each antenna boresight is pointed at the center of the sample. The Rx antennas are positioned in the far field with polarization parallel to the E-field emitted from the illuminated sample unless stated otherwise. The antennas are connected to a 30 GHz Teledyne–Lecroy real-time oscilloscope via SMA cables. Estimates of the power emitted from the sample reported in the spectra are calculated using the Friis transmission equation; see Ref.^25^ for the power calculation for a single annulus. Average power spectra $\langle P\left(f\right)\rangle$ and the associated standard deviation are obtained by averaging over 20 identical trials. The reported spectra are normalized to the average total power measured by the antenna $P={\int }_{{f}_{0}}^{{f}_{1}}\langle P\left(f\right)\rangle df$ where ${f}_{1}$ and ${f}_{0}$ are the upper and lower frequency limits determined by the detection limits of the Rx antenna and SMA cable. These limits are used to calculate reported integrated normalized power values unless otherwise stated. The associated error bars represent the standard deviation of the integrated values over the 20 trials.

In-plane radiation patterns are obtained by positioning the Rx antenna around the sample at a constant distance and aligning the boresight of the antenna to the center of the sample. Radiation patterns are measured from ${\phi =0}^{^\circ }-{180}^{^\circ }$ due to the illumination geometry and spatial limitations on the positioning of the antennas. Average power values obtained at each angle are ratioed to the power measured by the stationary antenna positioned below the sample. The results are then normalized to the value along the $\phi ={0}^{^\circ }$ direction.

### Supplementary Information

Supplementary Information.

## Data Availability

Data underlying the results presented in this paper are not publicly available at this time but may be obtained from the corresponding author (T.J.B.) upon reasonable request.

## References

[1] Burford NM, El-Shenawee MO (2017). Review of terahertz photoconductive antenna technology. Opt. Eng..

[2] Bacon DR, Madéo J, Dani KM (2021). Photoconductive emitters for pulsed terahertz generation. J. Opt..

[3] Uematsu K, Maki K, Otani C (2012). Terahertz beam steering using interference of femtosecond optical pulses. Opt. Express.

[4] Zhu N, Ziolkowski RW (2013). Photoconductive THz antenna designs with high radiation efficiency, high directivity, and high aperture efficiency. IEEE Trans. TERAHERTZ Sci. Technol..

[5] Malhotra I, Jha KR, Singh G (2019). Beam steering characteristics of highly directive photoconductive dipole phased array antenna for terahertz imaging application. Opt. Quant. Electron..

[6] Shi, W. *et al.* High efficient broadband terahertz radiation generated by photoconductive antenna array. in *2019 44th International Conference on Infrared, Millimeter, and Terahertz Waves (IRMMW-THz)* (IEEE, 2019). 10.1109/IRMMW-THz.2019.8874249.

[7] Tonouchi M (1996). Terahertz emission study of femtosecond time-transient nonequilibrium state in optically excited YBa_2_Cu_3_O_7__−__δ_ thin films. Jpn. J. Appl. Phys..

[8] Hangyo M (1996). Terahertz radiation from superconducting YBa_2_Cu_3_O_7- δ_ thin films excited by femtosecond optical pulses. Appl. Phys. Lett..

[9] Hegmann FA (1995). Electro-optic sampling of 1.5-ps photoresponse signal from YBa_2_Cu_3_O_7__−__δ_ thin films. Appl. Phys. Lett..

[10] Lindgren M (1997). YBa_2_Cu_3_O_7__−__x_ thin-film picosecond photoresponse in the resistive state. IEEE Trans. Appl. Supercond..

[11] Lindgren M (1999). Intrinsic picosecond response times of Y–Ba–Cu–O superconducting photodetectors. Appl. Phys. Lett..

[12] Kadin AM, Leung M, Smith AD (1990). Photon-assisted vortex depairing in two-dimensional superconductors. Phys. Rev. Lett..

[13] Kadin AM, Johnson MW (1996). Nonequilibrium photon-induced hotspot: A new mechanism for photodetection in ultrathin metallic films. Appl. Phys. Lett..

[14] Fukui T, Moto A, Murakami H, Tonouchi M (2001). Distribution of optically-generated vortices in YBCO thin film strips. Phys. C Supercond..

[15] Fukui T, Murakami H, Tonouchi M (2002). Investigation of optical magnetic flux generation in superconductive YBCO strip-line. IEICE Trans. Electron..

[16] Tonouchi M (2005). Recent topics in high-Tc superconductive electronics. Jpn. J. Appl. Phys..

[17] Zeldov E, Amer NM, Koren G, Gupta A (1989). Nonbolometric optical response of YBa_2_Cu_3_O_7–δ_ epitaxial films. Phys. Rev. B.

[18] Zeldov E (1989). Optical and electrical enhancement of flux creep in YBa_2_Cu_3_O_7__−__δ_ epitaxial films. Phys. Rev. Lett..

[19] Bluzer N (1991). Temporal relaxation of nonequilibrium in Y–Ba–Cu–O measured from transient photoimpedance response. Phys. Rev. B.

[20] Johnson MW, Herr AM, Kadin AM (1996). Bolometric and nonbolometric infrared photoresponses in ultrathin superconducting NbN films. J. Appl. Phys..

[21] Jukna A, Taneda T, Sobolewski R (2004). Control of gigahertz antenna radiation using optically triggered Y–Ba–Cu–O superconducting microbridges. Supercond. Sci. Technol..

[22] Jukna A, Lisauskas V, Parseliunas J, Abrutis A, Plausinaitiene V (2004). Photoresponse studies of pulsed-current I>Ic biased YBa_2_Cu_3_O_7−δ_ thin films. Phys. C Supercond..

[23] Dolasinski, B. *et al.* Ultrafast photo response in superconductive isotropic radiators for microwave generation (ed. Vodopyanov, K. L.) 93470H (2015). 10.1117/12.2083505.

[24] Bulmer J (2015). Tunable broadband radiation generated via ultrafast laser illumination of an inductively charged superconducting ring. Sci. Rep..

[25] Bullard TJ (2019). Microwave antenna properties of an optically triggered superconducting ring. Supercond. Sci. Technol..

[26] Tonouchi M, Tani M, Wang Z, Sakai K, Wada N (1997). Novel terahertz radiation from flux-trapped YBa_2_Cu_3_O_7__−__δ_ thin films excited by femtosecond laser pulses. Jpn. J. Appl. Phys..

[27] Silveira ES, Nascimento DC, Tinoco-S AF, Pina MVP (2017). Design of microstrip antenna array with suppressed back lobe. J. Microw. Optoelectron. Electromagn. Appl..

[28] Stutzman W, Thiele G (2013). Antenna Theory and Design.

[29] Ozyuzer L (2007). Emission of coherent THz radiation from superconductors. Sci. Wash..

[30] Dobrovolskiy OV (2018). Microwave emission from superconducting vortices in Mo/Si superlattices. Nat. Commun..

[31] Hosseini M (2015). Tuning of the terahertz radiation frequency from superconductors. IEEE Trans. Appl. Supercond..

