# Supplementary material for: Directional microwave emission from femtosecond-laser illuminated linear arrays of superconducting rings
Source: Sci Rep. 2023 Oct 23;13:18043. doi: 10.1038/s41598-023-44751-x (PMC10593795; doi:10.1038/s41598-023-44751-x)
Supplement: Supplementary file 1 — Supplementary Information. [file 41598_2023_44751_MOESM1_ESM.docx]

Supplementary Information: Effects of Charging Coil on GHz Emission

Thomas J. Bullard^1^**^,^**^2,*^, Kyle Frische^3^, Charlie Ebbing^1,5^, Stephen J. Hageman^3^, John Morrison^3^, John Bulmer^1,6^, Enam A. Chowdhury^4^, Michael L. Dexter^3^, Timothy J. Haugan^1^, Anil K. Patnaik^3^

^1^ Air Force Research Laboratory, Wright Patterson AFB, OH 45433-7251

^2^UES, Inc. Dayton, OH 45432

^3^Air Force Institute of Technology, Wright Patterson AFB, OH 45433-7765

^4^The Ohio State University, Columbus, OH 43210

^5^UDRI-University of Dayton Research Institute, Dayton OH 45469

^6^National Research Council, Washington, D.C. 20001

*tbullard@vt.edu

To examine the effect of the charging coil on electromagnetic emission from the samples, we put a broadband printed circuit board antenna inside the coil as shown in Fig. S1(a). Due to constraints of the SMA cable, we are not able to position the antenna at the same height as the superconducting rings (i.e., at the bottom edge of the solenoid windings). Rather, the circuit board antenna sits at the bottom of the charging coil apparatus. Nevertheless, we examine the effect of the charging coil apparatus on the emission from the antenna. We connect the antenna to a signal generator and sweep the frequency from 2-18 GHz at steps of 0.5 GHz. We record the signal convolved with the effects of the coil. We then remove the coil and repeat the measurement. The measured spectra with and without the solenoid are shown in Fig. S1(b).


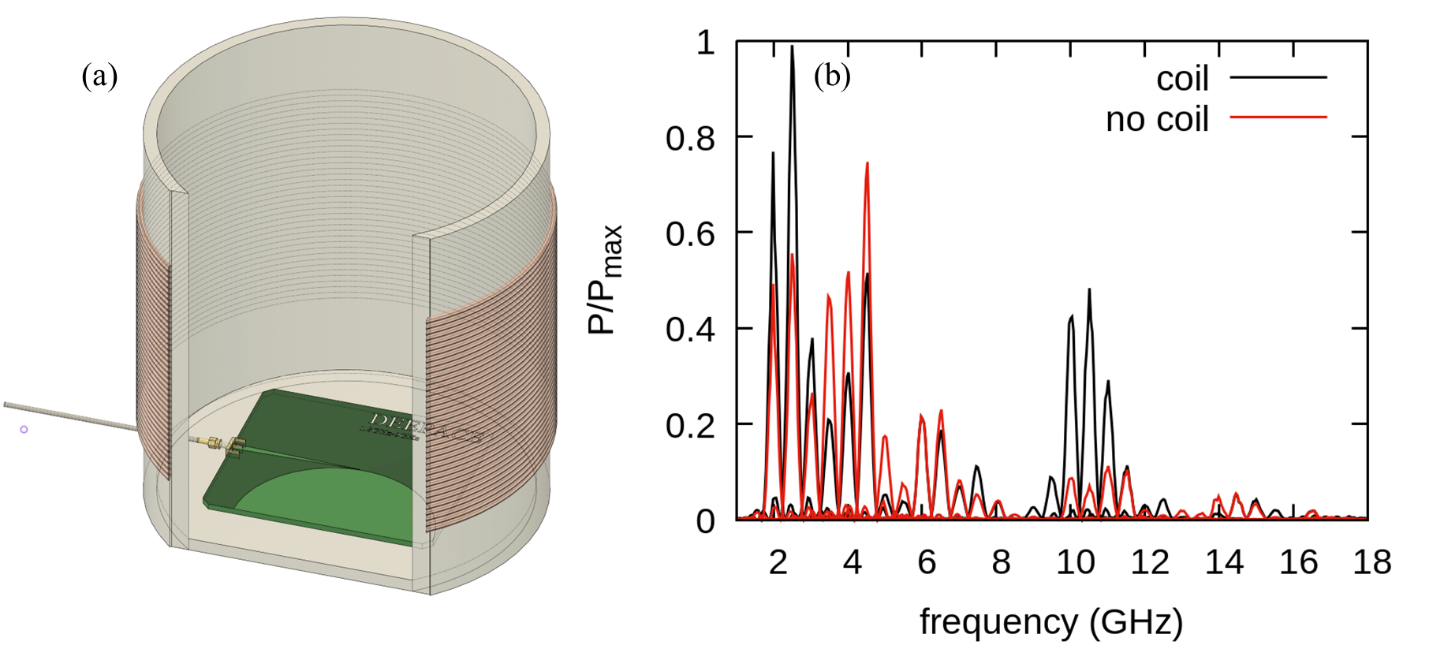


Fig. S1 (a) Broadband circuit board antenna seated inside and at the bottom of the charging coil. The radiating antenna is connected to a signal generator. (b) Spectra obtained below the antenna with and without the coil in place.

We define the angular and frequency dependent power ratio for the coil as, $G_{coil}\left( f,\phi\right)=\frac{P_{coil}\left( f,\phi\right)}{P_{no coil}\left( f,\phi\right)}$ where $P_{coil}\left( f,\phi\right)$ is the frequency and angle dependent power measured with the antenna inside the coil. $P_{no coil}\left( f,\phi\right)$ is the power from the antenna without the coil.

Results measured directly below and in the plane of the emitting antenna at $\phi=0^{^{\circ}}$ (directly opposite the SMA connector) are shown in Fig. S2. We note a peak detected below the coil at approximately 9.5 GHz as well as a smaller resonant peak in the plane of the antenna at 12 GHz. These features are observed in the spectra obtained with both the individual rings and arrays. If desired $G_{coil}\left( f,\phi\right)$ can be treated as an effective gain term in the Friis transmission calculations. However, when these effects are removed, we do not find any significant difference in radiation patterns reported in the Results section.


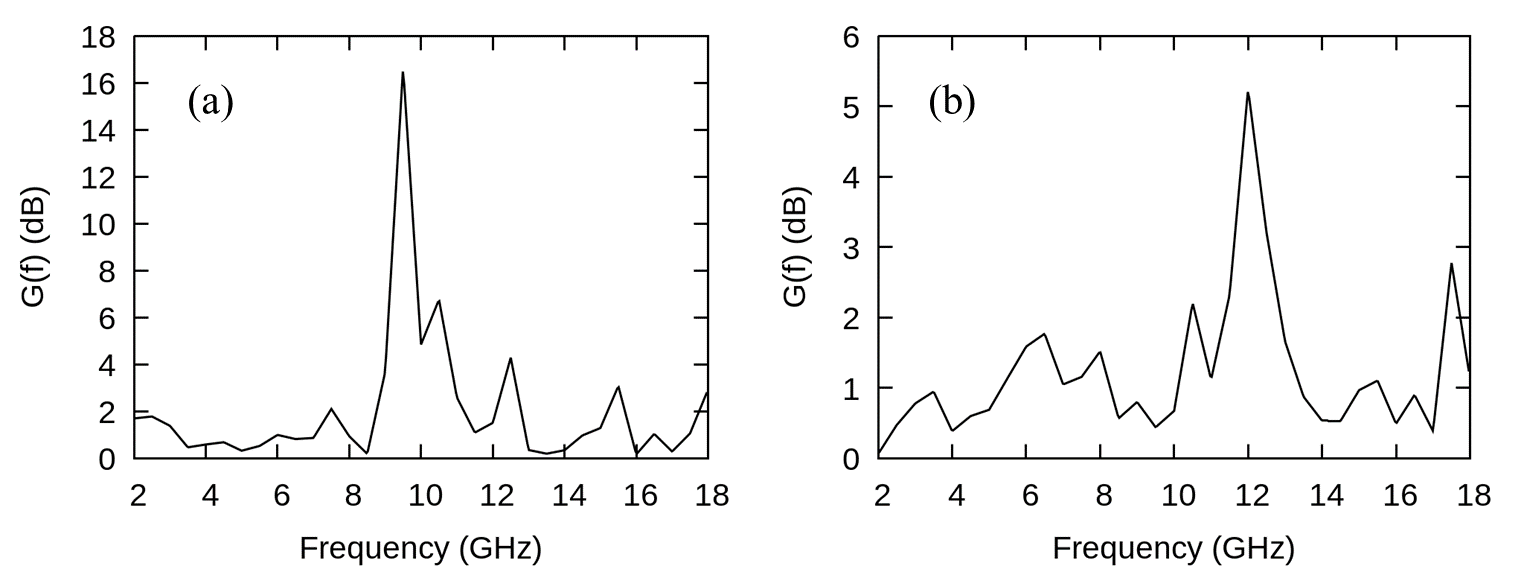


Fig. S2 (a) $G_{coil}\left( f \right)$measured below and (b) in the plane of the antenna. The in-plane measurement is along the $\phi=0^{^{\circ}}$ direction.


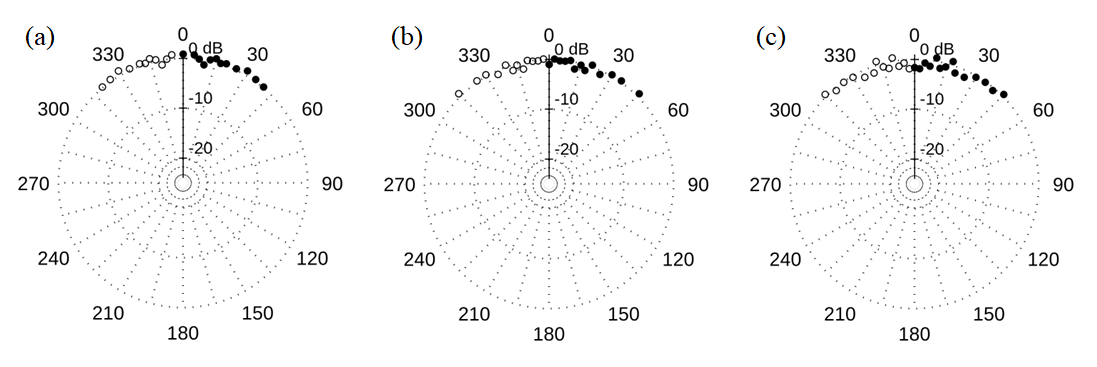


Fig. S3 $G_{coil}\left( \phi\right)$ in the plane of the ring for (a) 6.5 GHz, (b) 8.5 GHz and (c) 13.5 GHz. Data is measured from $0^{^{\circ}}-{45}^{^{\circ}}$. The mirror image is plotted as a guide for the eye.

Angular dependent effects of the coil are plotted in Fig. S3. Here, we examine $G_{coil}\left( \phi\right)$ for three frequencies: 6.5 GHz, 8.5 GHz, and 13.5 GHz. We find that $G_{coil}\left( \phi\right)$ does not greatly vary for angles between $0^{^{\circ}}-{45}^{^{\circ}}$. We find that the coil does not introduce any noticeable directivity to the radiation pattern, supporting the conclusion that the reported radiation patterns in the Results section are due to the array geometry and not the coil.
